# Notes and News

**Published:** 1898-09-03

**Authors:** 


### NOTES AND NEWS.

(Collected and Communicated.)

It is with much regret that we announce the death of Dr. Elizabeth Phillips, one of the three Europeans who, within the last few days, have fallen a victim to the plague in India. Despite all efforts to stop the spread of the fell disease, it is still greatly on the increase, over 2,300 deaths having occurred last week in the Bombay Presidency.

There is a certain amount of humour in the fact that the Jenner Memorial is being pushed at the very time when vaccination has received such a rebuff at the hands of the Government. The public appeal is for £100,000, to be handed over to the trustees of the Institute of Preventive Medicine, on the Chelsea Embankment, and it is asked that the memorial shall bear the name of the Jenner Institute. Some very large donations have been already received, and in face of the indignation at the present time felt by many strong believers in vaccination, the movement may now receive the required impetus to ensure its successful achievement.

The *London Gazette* publishes the new arrangements as to the rank and title of the medical officers of the Indian Medical Service who, so far as military rank and titles are concerned, are now placed on the same footing as their brethren of the British army. The changes to be made are as follows:—

| Present Ranks. | New Ranks. |
|---|---|
| Surgeon-Colonel ... ... | Colonel. |
| Brigade Surgeon-Lieutenant-Colonel ... ... ... | Lieutenant-Colonel. |
| Surgeon-Lieutenant-Colonel | Lieutenant-Colonel. |
| Surgeon-Major ... ... | Major. |
| Surgeon-Captain ... ... | Captain. |
| Surgeon-Lieutenant ... | Lieutenant. |

Officers above the rank of Surgeon-Colonel shall in future be styled Surgeon-Generals (ranking as Major-Generals), and the title of Surgeon-Major-Generals now serving shall be altered accordingly.

The following alterations are also to be made in the honorary ranks of the Senior Assistant Surgeons of the Subordinate Medical Department in India:—

| Present Honorary Ranks. | New Honorary Ranks. |
|---|---|
| Surgeon-Major ... ... | Major. |
| Surgeon-Captain ... ... | Captain. |
| Surgeon-Lieutenant ... | Lieutenant. |

Apropos of the conscience clause in the Vaccination Bill, it is not surprising that it has dawned upon the public mind that it has a distinct bearing upon the doings of the Peculiar People. The followers of this sect are as genuinely convinced that human interference with the Divine purpose is harmful as the bigotted anti-vaccinationist is of the harmful result of vaccination.

The hospital arrangements for the Soudan expedition have been carefully organised. In the front there are five fully-equipped field hospitals for each brigade. The sick will be passed down to a mud hospital at Atbara containing 200 beds, whence they will be carried by hospital trains to Wady Halfa. There is also a hospital for 200 beds at Abadia, below Berber, to which serious cases can be removed direct by boat from the front, so as to avoid the long railway journey. There are at present 58 British medical officers with the troops and on the line of communication. According to the special correspondent of the *Times* the sick rate among the troops at present does not exceed 3·3 per cent.

The latest news of the epidemic of typhoid fever at Belfast is not of a very satisfactory nature, though accounts of the steps taken to meet the contingency are, happily, reassuring. The prompt measure of the Most Rev. Dr. Henry, in suggesting that the wards of the Mater Hospital should be thrown open for the admission of typhoid patients, has earned the gratitude of the entire community. When the report of the serious condition of the overcrowding of hospitals by typhoid cases reached the Rev. Dr. Henry, who was away on a brief holiday, he immediately wired directions that all wards of the Mater Infirmorum Hospital available for the reception of patients should be opened at once for the reception of sufferers, without distinction of creed.

A regrettable occurrence appears to have taken place at the Royal Infirmary, Glasgow, towards the beginning of August, when a young assistant doctor was "ducked" in a bath of cold water after a mock trial by his fellow house doctors. As the matter is apparently going to be settled in the police court, the true facts of the case will doubtless be forthcoming shortly, but in the meantime it appears that the victim gave umbrage to his colleagues by refusing to receive in the wards under his charge certain cases which he considered should be treated elsewhere. It is difficult to believe that such can have been the only reason for resorting to such extreme measures, and presumably more light will be thrown on the matter when the case comes into court. The assailants, it is said, tendered an apology when they saw their victim intended to carry the matter further, but not until the time within which an apology had been demanded had passed. Such trials and punishments by "subaltern courts martial" are not unknown in the army, but the offences dealt with by them are social and not professional, and without attempting for one moment to justify such drastic treatment for social offences, it is very obvious that a similar method of expressing disapprobation of the manner in which an official carries out his duties comes under quite a different category.

